# Agranular frontal cortical microcircuit underlying cognitive control in macaques

**DOI:** 10.3389/fncir.2024.1389110

**Published:** 2024-03-27

**Authors:** Beatriz Herrera, Jeffrey D. Schall, Jorge J. Riera

**Affiliations:** ^1^Department of Biomedical Engineering, Florida International University, Miami, FL, United States; ^2^Centre for Vision Research, Centre for Integrative & Applied Neuroscience, Department of Biology and Psychology, York University, Toronto, ON, Canada

**Keywords:** agranular frontal cortex, macaques, cognitive control, microcircuit, EEG biomarkers

## Abstract

The error-related negativity and an N2-component recorded over medial frontal cortex index core functions of cognitive control. While they are known to originate from agranular frontal areas, the underlying microcircuit mechanisms remain elusive. Most insights about microcircuit function have been derived from variations of the so-called canonical microcircuit model. These microcircuit architectures are based extensively on studies from granular sensory cortical areas in monkeys, cats, and rodents. However, evidence has shown striking cytoarchitectonic differences across species and differences in the functional relationships across cortical layers in agranular compared to granular sensory areas. In this minireview, we outline a tentative microcircuit model underlying cognitive control in the agranular frontal cortex of primates. The model incorporates the main GABAergic interneuron subclasses with specific laminar arrangements and target regions on pyramidal cells. We emphasize the role of layer 5 pyramidal cells in error and conflict detection. We offer several specific questions necessary for creating a specific intrinsic microcircuit model of the agranular frontal cortex.

## 1 Introduction

Cognitive control involves suppressing automatic or impulsive actions and monitoring errors for successful goal-directed behavior. Phenomenological models of cognitive control formulate this function as two competitive action plans that must be resolved to achieve correct performance (Botvinick et al., 2001). Performance monitoring and executive control can be investigated using the stop-signal task (Verbruggen and Logan, 2009). Electrophysiological studies in human and non-human primates have described the scalp potentials associated with performance monitoring, the error-related negativity or ERN for error detection, and the N2 component for conflict detection (Gehring et al., 2012; Sajad et al., 2022). While the timing and amplitude of these event-related potentials are useful biomarkers of neurological disorders (Bates et al., 2002; Xiao et al., 2011; Foti et al., 2012; Balogh et al., 2017; Gorka et al., 2017; Moser, 2017; Marquardt et al., 2018; Riesel, 2019), studying the underlying intrinsic microcircuit mechanisms is essential to understand the pathology indicated by the biomarkers.

Insights about neocortical intrinsic microcircuit mechanisms have resulted in the so-called canonical cortical microcircuit (CCM) (Gilbert and Wiesel, 1983; Douglas et al., 1989). In general, the CCM consists of 3 layers – a supragranular (L2/3), a granular (L4), and an infragranular (L5/6) layer – comprised of excitatory and inhibitory neuronal populations, uniformly distributed across layers (Gilbert and Wiesel, 1983; Douglas et al., 1989). Feedforward inputs arrive at the granular layer, targeting spiny stellate cells (SSCs), other L4 neurons, and pyramidal cells (PCs) with dendrites in this layer (Gilbert and Wiesel, 1983; Silberberg et al., 2005). SSCs send feedforward connections to the supragranular PCs (Gilbert and Wiesel, 1983; Silberberg et al., 2005). L3 PCs are heavily interconnected and project to L2 PCs (Silberberg et al., 2005). Supragranular PCs receive inputs and provide output to associational brain regions (Silberberg et al., 2005). Information is then sent from the supragranular PCs to the infragranular PCs, which send feedback projections back to the granular layer (Gilbert and Wiesel, 1983; Douglas et al., 1989; Silberberg et al., 2005). Infragranular L5 PCs consist of thick tufted L5 PCs that project to subcortical regions – the major source of neocortical output – and thin untufted L5 PCs that project to the contralateral hemispheres (Silberberg et al., 2005). Infragranular L6 PCs include corticothalamic and corticocortical PCs (Silberberg et al., 2005).

Different variants of the CCM have been proposed and adopted in the literature relying on the assumption that intrinsic cortical circuit architectures are homogenous throughout the cortex (Douglas et al., 1989; Jones et al., 2007; Bastos et al., 2012; Pinotsis et al., 2017). However, they are mainly based on studies in monkeys, cats, and rodents’ primary visual or somatosensory areas with a distinct layer 4. These areas exhibit substantial interlaminar inhibitory-to-excitatory connections (Beul and Hilgetag, 2015). L3 excitatory neurons in the primary visual cortex are inhibited by interneurons in layers 4 and 5, and L4 excitatory neurons are inhibited by interneurons in L5 (Kätzel et al., 2011; Beul and Hilgetag, 2015). In the primary somatosensory cortex, L4 interneurons inhibit L3 and L5 excitatory neurons, while L5 interneurons inhibit L4 excitatory neurons (Kätzel et al., 2011; Beul and Hilgetag, 2015). Inhibitory neurons either target the somata, perisomatic dendrites, and axon initial segment (large, nest, and small basket cells and chandelier cells), affecting the action potential generation, or the dendritic domain (mid-range and proximal dendrites: bitufted, double-bouquet, bipolar and neurogliaform cell; and distal dendrites: Martinotti cells), influencing local dendritic and coincident detection integration (Markram et al., 2004; Silberberg et al., 2005).

Different studies have demonstrated that the ERN and N2-component originate from medial frontal areas such as Supplementary Eye Field (SEF), an agranular area cytoarchitecturally within area F7 of macaque monkeys, and Anterior Cingulate Cortex (ACC), agranular areas without a well-defined layer 4 (Stuphorn et al., 2000; Garavan et al., 2003; Ito et al., 2003; Emeric et al., 2008, 2010; Gehring et al., 2012; Scangos et al., 2013; Sajad et al., 2019; Fu et al., 2023). Multiple studies have reported variations in cytoarchitectonic differentiation across the cortex and functional differences in the relationship across cortical layers in agranular areas compared to granular sensory areas (Godlove et al., 2014; Beul and Hilgetag, 2015; Ninomiya et al., 2015; Wagstyl et al., 2020).

We will update these proposals in three ways: a) incorporate recent findings by our group about the role of a subset of PCs, b) incorporate laminar organization of interneurons according to Ca^2+^-binding immunohistological data recorded and modeled by our labs, and c) evaluate the impact of these microcircuit organization on large-scale EEG.

In this minireview, we present a tentative microcircuit model of the agranular frontal cortex of macaques based on a current literature survey. The proposed model incorporates recent experimental and theoretical findings. It delineates the laminar arrangement of GABAergic interneurons subclasses in the macaque frontal cortex and their connectivity with PCs. Lastly, we discuss the limitations of the available literature.

## 2 Microcircuit of agranular frontal cortex

**Figure 1:**
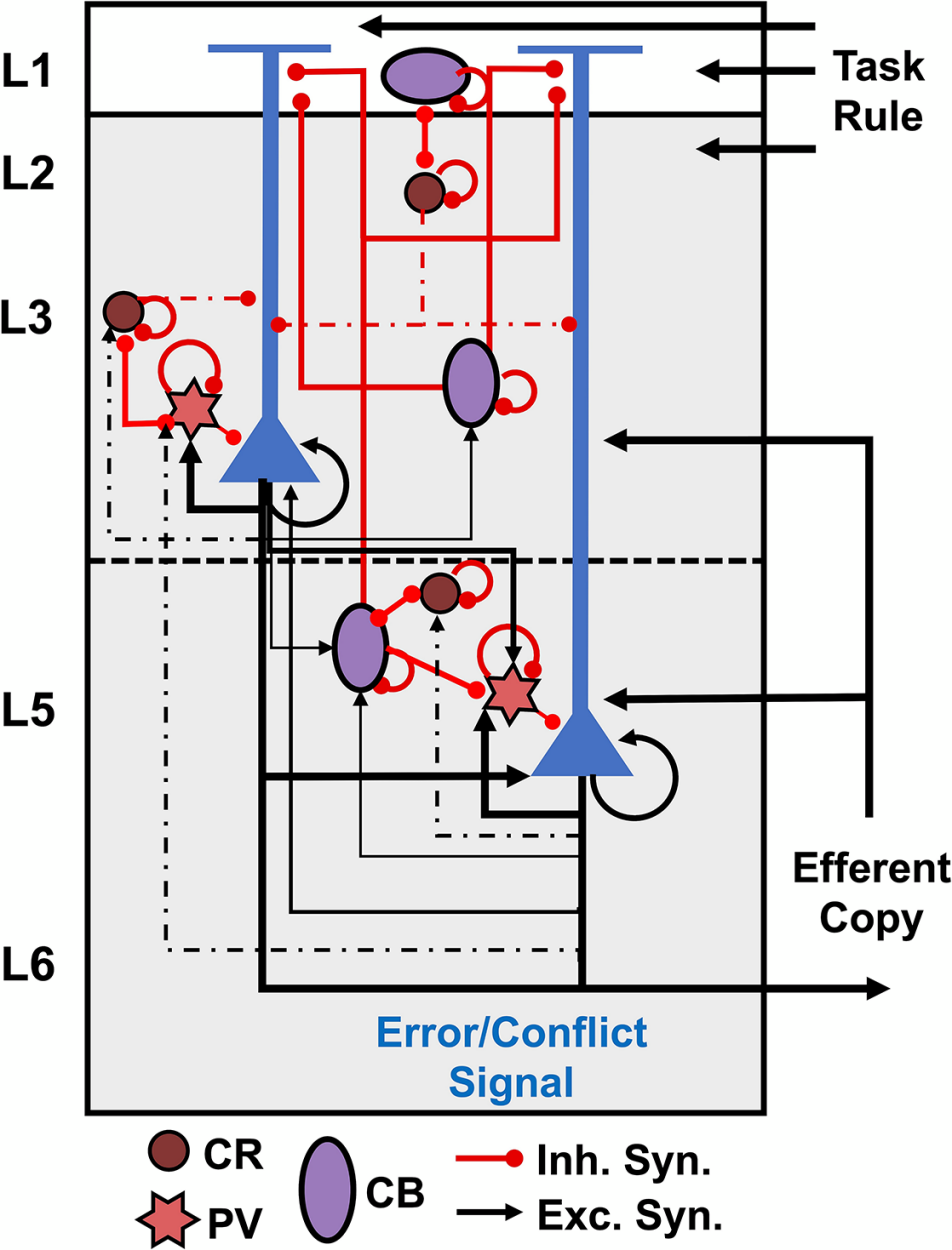
Cortical microcircuit for agranular frontal cortex. Pyramidal cells are shown in blue, and interneurons are color-coded according to the calcium-binding proteins PV – parvalbumin (start), CB – calbindin (circle), and CR – calretinin (oval). Excitatory connections are represented by black arrows, and red lines with a dot ending represent inhibitory connections.

Figure 1 summarizes our conjecture of the cortical microcircuit for the agranular frontal cortex. Recent microcircuit processing models have suggested that conflict detection can be achieved by coincidently detecting synaptic inputs by L5 PCs in the medial frontal cortex (Alexander and Brown, 2011; Cohen, 2014; Dembrow et al., 2015). Sajad et al. (2019) suggested that L5 PCs receive coincident inputs representing an efferent copy of the motor command from the mediodorsal thalamus and the task rule from the prefrontal cortex. Our recent study demonstrated that PCs monitor errors via increased excitatory input with branch-specific encoding in apical dendrites with intrinsic theta rhythms (Herrera et al., 2023). Hence, in our microcircuit model, external inputs representing the task rule arrive on the distal apical dendrites of PCs, while those representing the efferent copy arrive on their proximal dendrites. These PCs are connected to inhibitory interneurons that regulate their excitation.

Evidence suggests GABAergic interneurons in the agranular cortex support more intra- than interlaminar inhibition (Gabbott and Bacon, 1996; Lewis et al., 2002; Kätzel et al., 2011; Beul and Hilgetag, 2015). GABAergic interneurons in macaques can be divided into three main populations according to the calcium-binding proteins: PV – parvalbumin, CB – calbindin, and CR – calretinin (Godlove et al., 2014; Medalla et al., 2023), as depicted in Figure 1. The laminar arrangement of the interneurons was determined based on histological data of interneuron populations reported for SEF (Godlove et al., 2014; Herrera et al., 2023). In primates, CR interneurons are most commonly bipolar cells, analogous to bipolar VIP+ interneurons in rodents, bitufted cells, and double bouquet cells (Gabbott and Bacon, 1996; DeFelipe, 1997; Markram et al., 2004; Zaitsev et al., 2005; Džaja et al., 2014; Tremblay et al., 2016; Medalla et al., 2023). CB interneurons are analogous to rodent somatostatin (SST) interneurons – double bouquet and Martinotti cells – and also include neurogliaform cells (Gabbott and Bacon, 1996; DeFelipe, 1997; Markram et al., 2004; Tremblay et al., 2016; Medalla et al., 2023). PV interneurons consist of multipolar basket cells and chandelier cells (Gabbott and Bacon, 1996; DeFelipe, 1997; Markram et al., 2004; Tremblay et al., 2016; Medalla et al., 2023).

CR interneurons mainly synapse onto the dendrites of other GABAergic interneurons, providing strong innervation onto other CR cells and SST interneurons (Lewis et al., 2002; Melchitzky and Lewis, 2008; Džaja et al., 2014). They also target, but with a lower density, the dendrites of PCs synapsing primarily onto the dendritic shafts rather than the dendritic spines of PCs (Lewis et al., 2002; Melchitzky and Lewis, 2008). CR bipolar and bitufted cells target PCs’ proximal and middle dendritic regions (Džaja et al., 2014; Medalla et al., 2023). CR double bouquet cells project to the basal dendrites of PCs (Zaitsev et al., 2005; Džaja et al., 2014).

CB SST interneurons directly inhibit the apical dendrites of PCs (Džaja et al., 2014; Medalla et al., 2023). SST Martinotti cells form wide axonal arbors that extend up to 800 μm, inhibiting the apical tuft of PCs (Zaitsev et al., 2005; Medalla et al., 2023). CB neurogliaform cells have axons mostly confined within L2-3 that target the dendrites and spines of PCs in these layers (Zaitsev et al., 2005; Medalla et al., 2023). They also target the dendrites of other GABAergic interneurons but at a lower rate than CR interneurons (Melchitzky and Lewis, 2008).

PV chandelier cells only inhibit the axon initial segment of PCs, whereas PV basket cells target the soma and proximal dendritic shafts and spines of PCs (Lewis et al., 2002). PV neurons in L2-3 receive more excitatory inputs from local PCs than CR interneurons (Melchitzky et al., 1998, 2001; Lewis et al., 2002; Melchitzky and Lewis, 2003). About 53% of local axon terminals from L3b PCs oppose PV interneurons within 300μm of their soma, whereas only 5% target CR interneurons (Melchitzky and Lewis, 2003). Approximately 34 and 25% of L2-3a PCs axon terminals oppose the dendritic shafts of PV and CR interneurons, respectively (Melchitzky and Lewis, 2003). Overall, about 50% of local axon terminals from L2-3 PCs in monkey prefrontal cortex target equally the dendritic spines of other PCs and the dendritic shafts of nearby GABAergic interneurons (Melchitzky et al., 1998, 2001; Lewis et al., 2002; Melchitzky and Lewis, 2003). This evidence suggests PCs in these layers potentially target CB cells (Table 1 – Question 1).

**Table 1:** Outstanding questions.

What are the patterns of intrinsic intralaminar connections between L2-3 pyramidal cells and CB interneurons in the agranular frontal cortex?
What are the patterns of interlaminar pyramidal cell connectivity in agranular frontal cortex?
What are the patterns of intrinsic intralaminar pyramidal cell connectivity in L5-6 of agranular frontal cortex?
What is the role of GABAergic interneurons in the agranular frontal cortex in midfrontal theta genesis?
What neurotransmitter receptors are expressed in each neuron type in the agranular frontal cortex?
Do synaptic currents with slow kinetics help drive EEG rhythmogenesis?
Do intrinsic inter- and intralaminar connections in the agranular frontal cortex of macaque monkeys extend to other agranular frontal areas?
Which ionic channels are expressed in distinct neurons in agranular frontal cortex? Do they have the same kinetics as those in rodents?

L2 and superficial L3 PCs primarily project within these layers with descending axons emitting collaterals in L5 (Levitt et al., 1993). Deep L3 PCs generate significant horizontal projections with periodic terminations in L1-3 and project to L5-6 (Levitt et al., 1993). L5 PCs extensively project from L5 to L6 and terminate diffusely in L1-3 (Levitt et al., 1993). However, we found no information about the specific patterns of interlaminar connectivity between PCs in superficial and deep layers nor about the intralaminar PC connectivity in deep layers (Table 1 – Questions 2 and 3).

## 3 Midfrontal theta genesis

An increase in midfrontal theta has been associated with cognitive control tasks in human and non-human primates’ EEG studies (Cavanagh and Frank, 2014; Cohen, 2014; Herrera et al., 2023). We recently demonstrated that the observed transient increase in theta power on error versus correct trials arises from conflict detection in L5 PCs (Herrera et al., 2023). Additionally, we found that in contrast to previous hypotheses (Cohen, 2014), L5 PCs generate intrinsic theta oscillations that are only visible in the local field potentials and EEG after phase-reset by synchronized external stimuli (Herrera et al., 2023). Multiple studies have reported that L5 PC theta-band resonance is disrupted by pharmacological blockage of HCN1 (Ih) channels (Dickson et al., 2000; Giocomo and Hasselmo, 2009; Colgin, 2013; Neymotin et al., 2013; Stark et al., 2013). Our circuit suggests L5 PC theta-band resonance may be regulated by PV cells, which exert direct inhibition onto these. They could provide the synchronized input needed to reset the ongoing subthreshold theta oscillations in L5 PCs. Furthermore, in agreement with Cohen (2014) hypothesis, CB SST interneurons may facilitate theta-band resonance in these neurons by hyperpolarizing their apical dendrites and, as a result, activating the Ih channels. At the same time, CR interneurons may influence both phenomena via disynaptic inhibition. Theoretical studies are necessary to test these hypotheses (Table 1 – Questions 4).

Theta oscillations have also been linked to NMDA and “slow” GABA-A receptors (Buzsáki, 2002; Moolchand et al., 2022), receptors densely expressed in the medial prefrontal cortex of macaques (Rapan et al., 2021). However, our biophysical model of L3 error PCs under AMPA and NMDA excitatory synaptic inputs did not show intrinsic theta oscillation as the L5 error PC model (Herrera et al., 2023). Further theoretical studies evaluating the role of synaptic currents with slow kinetics, such as “slow” GABA-A and GABA-B receptors, are needed to examine the influence of GABAergic interneurons in EEG rhythmogenesis (Table 1 – Questions 5 and 6).

## 4 Implications for EEG biomarkers

Multiple studies have shown that impaired cognitive control EEG event-related potentials (ERP) are a biomarker of psychiatric disorders such as ADHD (Balogh et al., 2017; Marquardt et al., 2018), OCD (Xiao et al., 2011; Riesel, 2019), schizophrenia (Bates et al., 2002; Foti et al., 2012), and anxiety (Gorka et al., 2017; Moser, 2017). Theoretical studies have shown that variants of schizophrenia-associated genes affect the nonlinear integrative properties in L5 PCs, impairing their coincident detection capabilities (Mäki-Marttunen et al., 2016, 2019). Our previous studies demonstrated a larger contribution of L5 PCs to the EEG signals because of the nonlinear integration between their proximal and distal apical dendritic regions (Herrera et al., 2022, 2023). Additionally, they can act as pacemakers of neocortical theta oscillations, another biomarker of cognitive control (Nigbur et al., 2011; Cavanagh and Frank, 2014; Cavanagh, 2015). Impairment of their nonlinear dendritic dynamics could result in an ERP with reduced magnitude and a decrease in midfrontal theta power. Morphological alterations of PCs (lower dendritic spine density and soma size) in superficial layers have also been reported in dorsomedial prefrontal cortex of subjects with schizophrenia (Lewis et al., 2012; Schoonover et al., 2020). This causes lower recruitment of PV cells, disrupting EEG gamma oscillations, which reflects deficits in cognitive control (Lewis et al., 2012; Schoonover et al., 2020).

On the other hand, OCD has been associated with increased ERN amplitude (Xiao et al., 2011; Riesel, 2019). Evidence suggests the increase in magnitude is caused by increased glutamatergic neurotransmission, leading to persistent activity in prefrontal areas (Rosenberg et al., 2004; Pittenger et al., 2006). Prefrontal cortex persistent activity arises from the recurrent network of PCs and interneurons and depends on the interplay of the slow kinetics NMDA and GABA-B currents (Wang, 1999; Papoutsi et al., 2013; Konstantoudaki et al., 2014; Curtis and Sprague, 2021). This enhanced PC activity would lead to larger intracranial brain sources and, as a result, a larger ERP amplitude.

Establishing a microcircuit model of the Agranular Frontal Cortex is imperative to understanding how changes in macroscopic ERPs translate into changes in microcircuit processing. Our circuit model offers a first step toward this goal.

## 5 Discussion

We presented a first-draft microcircuit of agranular frontal cortex based on a literature survey and a previous histological study from our group (Godlove et al., 2014). This offers a first attempt to create a complete microcircuit model for agranular frontal cortex of macaques, providing a powerful tool to inform computational modeling studies in macaques. Yet, it is far from complete and depends on many untested assumptions (Table 1). Further studies in agranular areas of the macaque frontal cortex are needed to demonstrate the validity of this circuit model and gain more insights into the inter- and intra-laminar connections. Most of the available literature about the intrinsic microcircuit of the macaque frontal cortex was based on studies in granular prefrontal areas (e.g., area 9 and 46, and dorsolateral prefrontal cortex or dlPFC) (Melchitzky et al., 1998, 2001; Lewis et al., 2002; Melchitzky and Lewis, 2003; Medalla et al., 2023). They offered valuable information compared to the available rodent literature, but there could be differences in the intrinsic connectivity patterns compared to agranular areas, leading to Question 7 in Table 1.

Additionally, these studies focused on the intrinsic circuit of superficial layers (L1-3), providing limited information about intralaminar connectivity patterns or the connectivity patterns within deep layers (L5-6) (Table 1 – Questions 2 and 3). Hence, we considered the same within laminar connectivity patterns in deep and superficial layers. Furthermore, studies assessing the differential targeting of GABAergic interneurons by PCs only studied the CR and PV interneuron subclasses (Melchitzky et al., 1998, 2001; Lewis et al., 2002; Melchitzky and Lewis, 2003, 2008) (Table 1 – Questions 1). To move the field forward, we need to address these questions and develop a biophysical computational model of the agranular frontal cortex microcircuit for creating testable hypotheses. To that end, we also need to perform more morphological characterizations of neurons in the agranular cortex and their ionic channels and kinetics (Table 1 – Question 8).

## Author contributions

BH: Conceptualization, Funding acquisition, Visualization, Writing – original draft, Writing – review & editing. JS: Conceptualization, Funding acquisition, Supervision, Writing – review & editing. JR: Conceptualization, Funding acquisition, Supervision, Writing – review & editing.
